# Hypomethylation of PRDM1 is associated with recurrent pregnancy loss

**DOI:** 10.1111/jcmm.15335

**Published:** 2020-04-29

**Authors:** Guizhen Du, Mingming Yu, Qiaoqiao Xu, Zhenyao Huang, Xiaomin Huang, Li Han, Yun Fan, Yan Zhang, Ruohan Wang, Shuyu Xu, Xiumei Han, Guangbo Fu, Shuyan Lv, Yufeng Qin, Xinru Wang, Chuncheng Lu, Yankai Xia

**Affiliations:** ^1^ State Key Laboratory of Reproductive Medicine Institute of Toxicology Nanjing Medical University Nanjing China; ^2^ Key Laboratory of Modern Toxicology of Ministry of Education School of Public Health Nanjing Medical University Nanjing China; ^3^ Huai‐An First Affiliated Hospital Nanjing Medical University Huaian China; ^4^ The First Clinical Medical College Nanjing Medical University Nanjing China; ^5^ Epigenetics & Stem Cell Biology Laboratory National Institute of Environmental Health Sciences Durham NC USA

**Keywords:** DNA methylation, gene expression, *PRDM1*, recurrent pregnancy loss, villus

## Abstract

Recurrent pregnancy loss (RPL) rates have continued to rise during the last few decades, yet the underlying mechanisms remain poorly understood. An emerging area of interest is the mediation of gene expression by DNA methylation during early pregnancy. Here, genome‐wide DNA methylation from placental villi was profiled in both RPL patients and controls. Subsequently, differentially expressed genes were analysed for changes in gene expression. Many significant differentially methylated regions (DMRs) were identified near genes dysregulated in RPL including *PRDM1*. Differentially expressed genes were enriched in immune response pathways indicating that abnormal immune regulation contributes to RPL. Integrated analysis of DNA methylome and transcriptome demonstrated that the expression level of *PRDM1* is fine‐tuned by DNA methylation. Specifically, hypomethylation near the transcription start site of *PRDM1* can recruit other transcription factors, like FOXA1 and GATA2, leading to up‐regulation of gene expression and resulting in changes to trophoblast cell apoptosis and migration. These phenotypic differences may be involved in RPL. Overall, our study provides new insights into *PRDM1*‐dependent regulatory effects during RPL and suggests both a mechanistic link between changes in *PRDM1* expression, as well as a role for *PRDM1* methylation as a potential biomarker for RPL diagnosis.

## 1 INTRODUCTION

Recurrent pregnancy loss (RPL), defined as more than two consecutive miscarriages, is viewed as a distinct disorder.^1^ It is estimated that 5% of women experience two clinical miscarriages and approximately 1% experience three or more losses.^2^ The aetiology of RPL is complicated, with known causal factors of RPL including genetic factors, anatomic abnormalities, autoimmune and endocrine factors.^3^ However, the aetiology of approximately half of RPL cases still remains unknown.^3^, ^4^, ^5^ During pregnancies, extravillous trophoblast cells invade the decidualized endometrium and remodel uterine spiral arteries to increase maternal blood flow to the placenta villi.^6^ The transitions from undifferentiated progenitors to differentiated trophoblast cells require dynamic epigenetic regulation,^6^ suggesting a role for DNA methylation as a potential mechanism regulating trophoblast differentiation. Despite numerous studies demonstrated that aberrant DNA methylation of genes is closely correlated with pregnancy loss, it remains unclear the roles of DNA methylome and transcriptome perturbations in RPL patients.^7^ In this study, we aimed to investigate the DNA methylome and transcriptome perturbations in RPL patients.

## 2 MATERIALS AND METHODS

### 2.1 Participating cohorts

This study was approved by the Institutional Ethics Committee of Nanjing Medical University. (detailed in the Supplementary Materials).

### 2.2 DNA isolation and bisulphite conversion

1μg DNA was used for bisulphite conversion with the EZ DNA methylation kit. (detailed in the Supplementary Materials).

### 2.3 Genome‐wide methylation profiling

Methylation data were processed using the R ChAMP package. (detailed in the Supplementary Materials).

### 2.4 RNA sequencing and analysis

RNA sequencing was done in Genesky. (detailed in the Supplementary Materials).

### 2.5 Bisulphite‐sequencing PCR

See Supplementary Materials.

### 2.6 Luciferase reporter assay

See Supplementary Materials.

### 2.7 Cell cycle, cell apoptosis and cell migration

See Supplementary Materials.

### 2.8 Chromatin immunoprecipitation (ChIP)

See Supplementary Materials.

### 2.9 Statistical analysis

See Supplementary Materials.

## 3 RESULTS

### 3.1 Differential DNA methylation pattern between recurrent pregnancy losses (RPLs) and control

**FIGURE 1:**
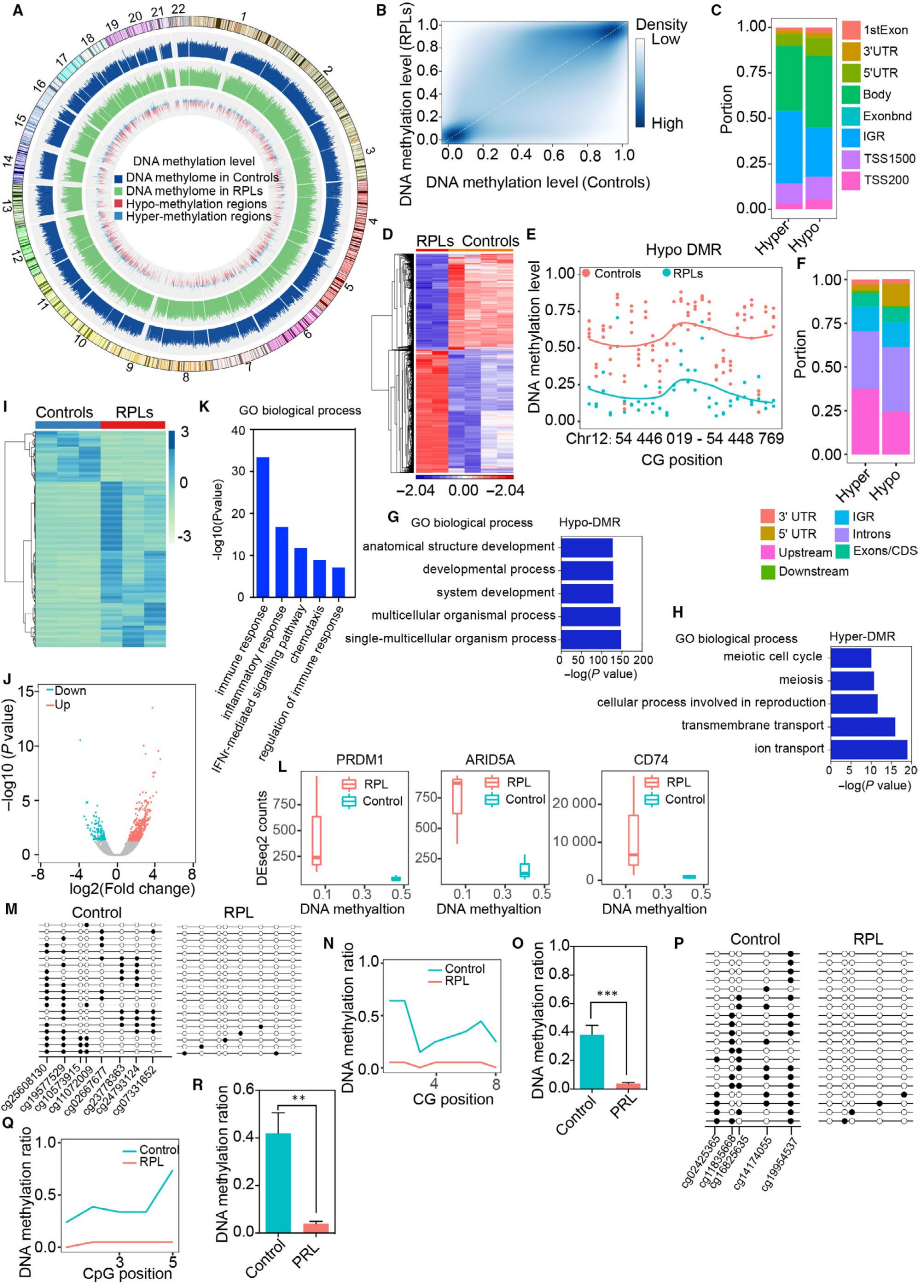
Recurrent pregnancy loss (RPL) shaped the DNA methylome and gene expressions in villus. (A) Circos plot of DNA methylation level in RPL and controls. (B) Correlation of DNA methylation level between RPL and controls. (C) Distribution of differential methylated cg probe in two groups. (D) Heatmap of differential methylated regions in RPL and controls. (E) Example of differential methylated regions. (F) Distribution of differential methylated regions in two groups. (G‐H) GO analysis of differential methylated regions in RPL and controls. (I) Heatmap of differential expressed genes in RPL and controls. (J) Volcano plot of differential expressed genes in RPL and controls. (K) GO analysis of differential expressed genes in RPL and controls. (L) Correlation between DNA methylation and gene expressions. (M‐O) BSP validation of DNA methylation level in PRDM1. (P‐R) BSP validation of DNA methylation level in ARID5A

We performed Illumina EPIC Bead Chip to investigate the differential DNA methylation between normal controls (n = 4) and RPL patients (n = 2). The overall methylation pattern was significantly different between cases and controls (Supplementary Material, Figure S1A, B). Evaluating genome‐wide DNA methylation level by β value, both site‐specific hyper‐ and hypomethylation was observed (Figure 1A,B). We totally observed 147 711 significant DMPs (Benjamini‐Hochberg adjusted *P* < .05, |Δβ| > 0.10), of which 39,274 CpGs were hypo‐methylated with the remaining 108,437 being hyper‐methylated. The majority of hypo‐methylated and hyper‐methylated CpG sites were enriched in gene body and intergenic region (IGR) (Figure 1C).

We used Bumphunter^8^ to identify the differentially methylated regions (DMRs) between RPL patients and controls. Totally, we generated a robust list of 3,830 DMRs with 1,688 hypo‐methylated regions and 2142 hyper‐methylated regions with FDR < 0.05 (Figure 1D; Supplementary Material, Table S3). An example of hypo DMR is displayed in Figure 1E. The distribution patterns of hyper‐ and hypo‐methylated DMRs were similar (Figure 1F). Functional annotation of genes near hypo‐methylated DMRs demonstrates enrichment for development signalling pathways (Figure 1G). Meanwhile, the hyper‐methylated DMRs were enriched in pathways related to embryo development (Figure 1H), suggesting DNA methylation during development is a fine‐tuning mechanism.

### 3.2 DNA methylation affected Gene Expressions in recurrent pregnancy losses (RPLs)

In order to assess whether DNA methylation changes affect the gene expression in RPLs, we carried out RNA‐seq in villus tissues from the patients with RPL (n = 3) and controls (n = 3). A total of 542 differentially expressed genes (DEGs) with fold change >2 and FDR <0.05 were identified using DESeq2 comparing RPL patients with controls (Figure 1I,J; Supplementary Material, Table S4). Top significant DEGs were randomly selected to validate using quantitative real‐time PCR, and the results were highly consistent (Supplementary Material, Figure S2A). Our results demonstrated that the DEGs were significantly enriched with genes involved in immune‐related pathways (Figure 1K).

To evaluate the influence of RPL‐related DNA methylation changes on gene expressions, we employed binding and expression target analysis (BETA).^9^ Hyper‐DMRs were significantly enriched near both up‐ and down‐regulated genes, indicating that DNA methylation may have dualistic effects on gene regulation, while hypo‐DMRs were only enriched in up‐regulated genes (Supplementary Material, Figure S2B,C). Correlations between DNA methylation and gene expression were shown at some DEGs (Figure 1L). To confirm the DNA methylation results, we performed bisulphite‐sequencing PCR (BSP) at *PRDM1* and *ARID5A* in 20 RPL patients and matched controls. Methylation of the DMR near *PRDM1* (Figure 1M‐O) and *ARID5A* (Figure 1P‐R) was lower (~5%) in RPLs than in controls (~40%). The data were highly consistent with the methylation array.

**FIGURE 2:**
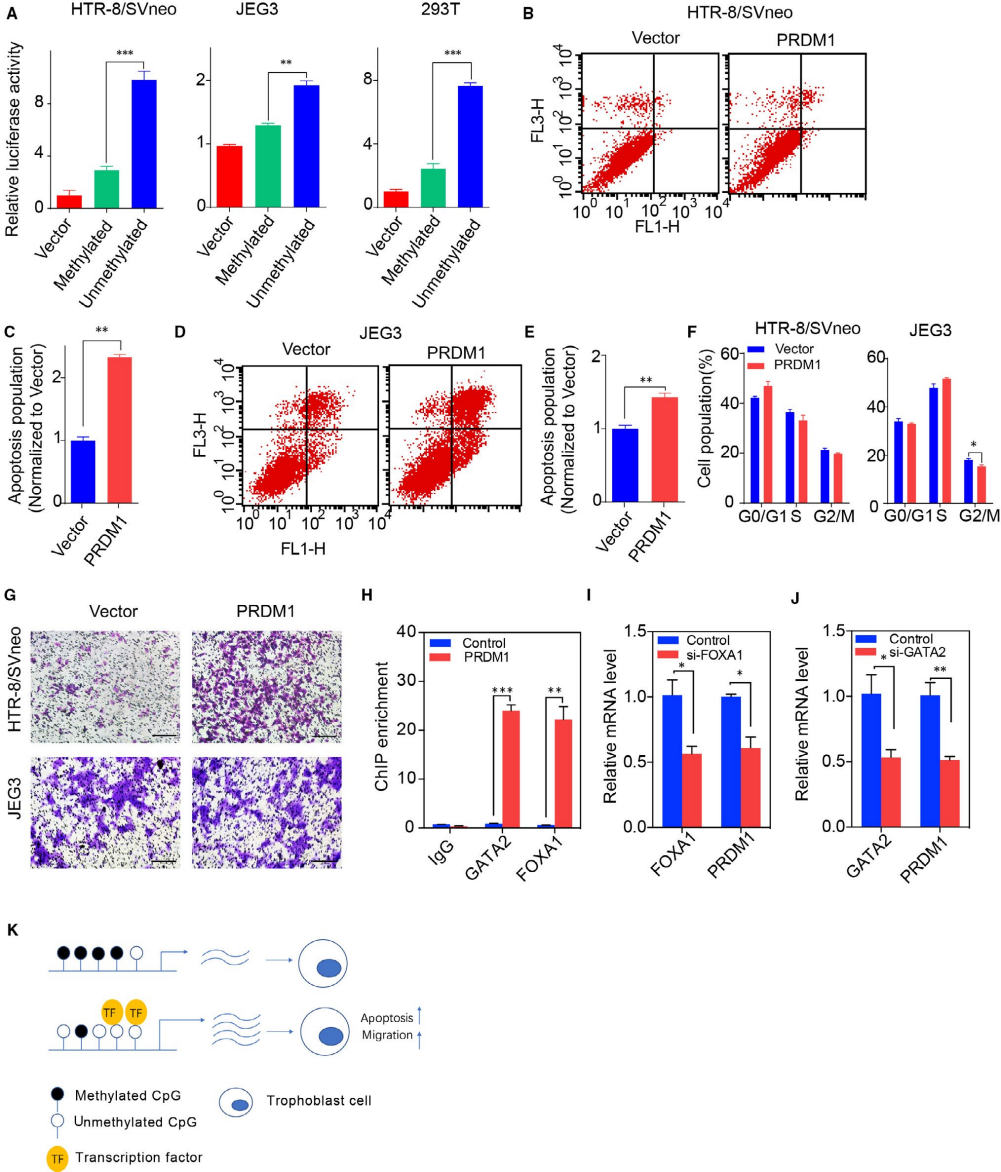
Effect of PRDM1 on cell apoptosis, cell cycle and migration and potential mechanisms. Luciferase activity of vector containing either methylated or unmethylated DMR of *PRDM1*. (B‐C) The percentage of apoptotic cells was increased significantly in HTR‐8/SVneo cell after over expressing *PRDM1*. (D‐E) The percentage of apoptotic cells was increased significantly in JEG3 cell after over expressing *PRDM1*. (F) PRDM1 affected cell cycle in JEG3 cell. (G) *PRDM1* affected the cell migration in HTR‐8/SVneo cell. Each data point represented the mean ± SE from three separate experiments in which treatments were performed in triplicate. *P < 0.05, **P < 0.01, **P < 0.001. (H) ChIP‐qPCR of GATA2 and FOXA1 showed significant enrichment in hypo‐methylated region of PRDM1. (I) Expression level of PRDM1 after FOXA1 knocked down. (J) Expression level of PRDM1 after GATA2 knocked down. (K)Proposed working model: hypo‐methylation induces the binding of GATA2 and FOXA1 in PRDM1, with a large induction of PRDM1 causing migration and apoptosis in trophoblast cells, leading to recurrent pregnancy loss

To further explore the DNA methylation responsible for the regulation of PRDM1, we carried out the dual‐luciferase reporter assay (Figure 2A). The reporter vector with either the methylated or the unmethylated insert was co‐transfected with an internal control (Renilla vector) into the human trophoblast cell line HTR‐8/SVneo, JEG‐3 and 293T cell line, respectively. Luciferase activities of unmethylated *PRDM1* DMR were significantly (*P* < .001) higher than that with the methylated insert in these three cell lines (Figure 2A).

### 3.3 *PRDM1 *DMR regulated trophoblast cells apoptosis and migration by recruiting *GATA2* and *FOXA1*

To determine whether *PRDM1* have effects on cell functions, we overexpressed *PRDM1* in HTR‐8/SVneo and JEG‐3 cell lines. As shown in Figure 2B‐E, apoptosis levels were significantly increased in the HTR‐8/SVneo (*P* < 0.01) and JEG‐3 cell lines (*P* < 0.01) after overexpressed *PRDM1*. Additionally, the high expression level of *PRDM1* delayed cell progression from S to M cell cycle transition in JEG‐3 cell line (*P* < 0.05) (Figure 2F). By transwell cell migration assays, we found that the number of cells migrating through transwell pores was significantly increased after *PRDM1* overexpression in HTR‐8/SVneo cell (Figure 2G).

In order to test whether there were other factors binding to the hypo‐methylated region of *PRDM1*, we used the online tool called PROMO to predict the putative transcription factor binding sites in DNA sequences based on TRANSFAC database.^10^, ^11^ We validated these binding sites by ChIP‐qPCR, and the results showed that FOXA1 and GATA2 significantly enriched in the hypo‐methylated region near *PRDM1* (Figure 2H). Knocking down FOXA1 and GATA2 by siRNA down‐regulated the expression level of PRDM1 (Figure 2I‐J). All these indicated that hypomethylation of *PRDM1* promoter could recruit GATA2 and FOXA1 binding and up‐regulate *PRDM1* expression in turn.

## 4 DISCUSSION

In the present study, we set out to identify changes in placental villi from recurrent pregnancy loss, when compared to healthy normal term placenta. PRDM1 was identified to be both significantly differentially methylated and expressed. To validate the functional effects of these changes, knockdown studies were performed in the placental cell lines, JEG‐3 and HRT8. These findings demonstrate that epigenetic regulation of PRDM1 is a potential aetiological factor for recurrent pregnancy loss.

PRDM1, a transcription regulator of cell fate in the embryo, is required for primordial germ cell specification and reprogramming of intestinal enterocytes.^12^ In our study, compared with controls, *PRDM1* was found to be expressed significantly higher in placental villi of RPL patients. All together, these results suggested that not only *PRDM1* deficiency, but also excess would both cause adverse effects on embryo development. Furthermore, we found that the DMR near transcription start site of *PRDM1* was hypo‐methylated. Hypomethylation in regulatory region will open the chromatin region and expose DNA sequence to transcription factors, which will recruit cofactors and affect the gene transcription.^13^ In this study, we found that hypomethylation of *PRDM1* could recruit GATA2 and FOXA1 binding. GATA2, a transcription factor in GATA family, was reported to regulating gene regulatory network during self‐renew and differentiation of the trophoblast cells.^14^ Specifically, others have shown that knockout Gata2/Gata3 led to embryonic lethality probably through impairing trophoblast development.^15^

Additionally, we identified major differences in immune genes in the transcriptomic analysis. Similar to previous findings that suggest disruption of immune tolerance will affect normal pregnancy, transcriptomic data for RPL patients were significantly changed in immune genes. Specifically, disturbance of immune balance induced pro‐inflammatory with highly expressed chemokines (CXCL8, CCL2) and adhesion molecules (ICAM1) in RPL^16^ was observed in our data.

In summary, we propose a model (Figure 2K) that hypomethylation at the *PRDM1* promoter induces the binding of GATA2 and FOXA1, leading to an induction of PRDM1 expression. This increase in expression results in increased migration and apoptosis in trophoblast cells, leading to RPL. Of importance, this suggests that the methylation level of PRDM1 may be a promising target for intervention in RPL as well as a promising biomarker for early diagnosis of RPL.

## CONFLICT OF INTEREST

The authors declare that there are no conflicts of interest associated with this study.

## AUTHORS' CONTRIBUTIONS

XW, CL and YX directed the study, obtained financial support and were responsible for study design. GD, MY, QX and ZH performed experiments. MY and YQ analysed data. GD, MY and YQ wrote the manuscript. XH, LH, YF, YZ, RW, SX, XH, GF and SL provided reagents and processed tissue samples. All authors read and approved the final manuscript.

## Supporting information

Fig S1‐S2

Table S1

Table S2

Table S3

Table S4

Supplementary Material

## Data Availability

The data used to support the findings of this study are available from the corresponding author upon request.
