# Supplementary material for: Hypomethylation of PRDM1 is associated with recurrent pregnancy loss
Source: J Cell Mol Med. 2020 Apr 29;24(12):7072–7. doi: 10.1111/jcmm.15335 (PMC7299696; doi:10.1111/jcmm.15335)
Supplement: Supplementary file 1 — Fig S1‐S2 [file JCMM-24-7072-s001.docx]

**Figure S1. Characterization of CpG methylation patterns in RPL and controls**

(A) Density plot of genome-wide DNA methylation levels in RPL and controls. (B) Sample clustering based on DNA methylation data in two groups.

**
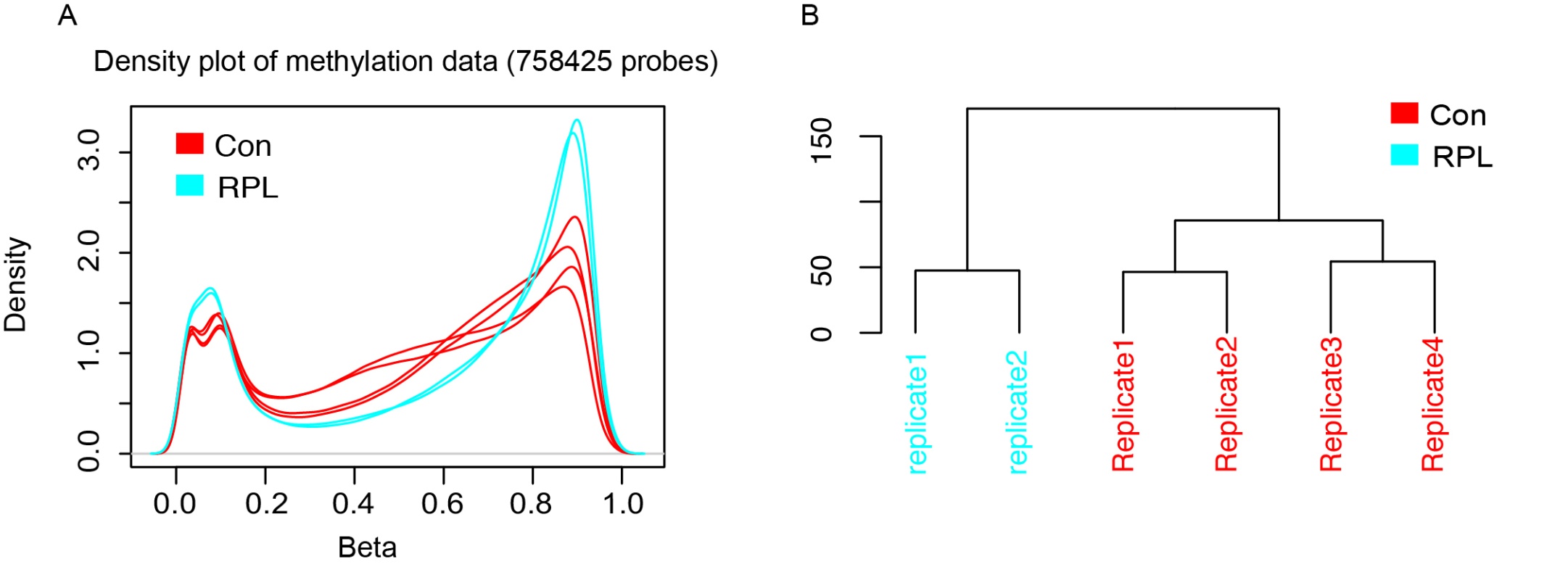
**

**Figure S2. RPL-related DMRs are significantly correlated with DEGs**


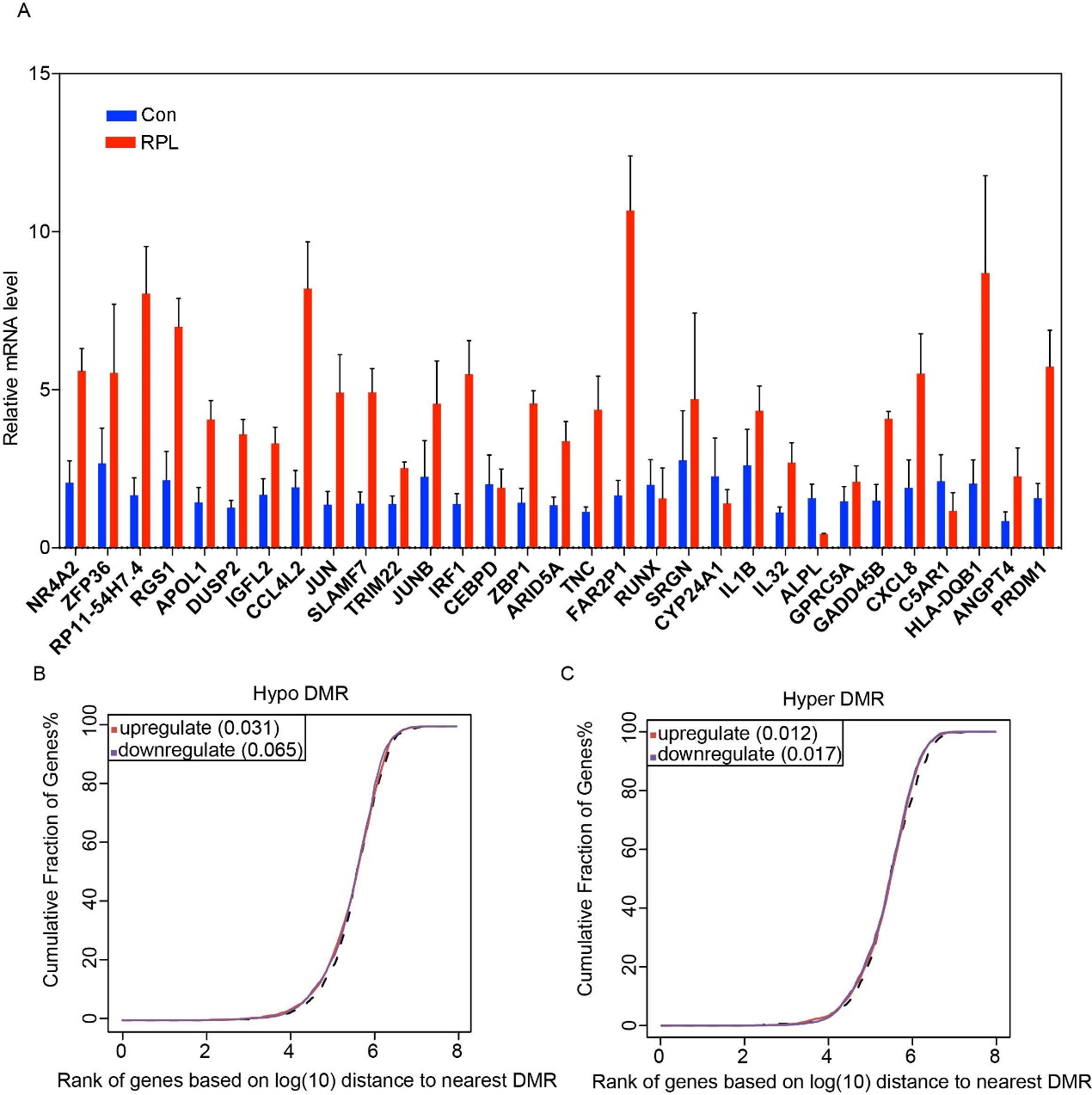
(A) qRT-PCR results of selective DEGs identified in RNAs-seq. (B) BETA with hypo or hyper-DMRs and differential gene expression data.
