# Supplementary material for: Hypomethylation of PRDM1 is associated with recurrent pregnancy loss
Source: J Cell Mol Med. 2020 Apr 29;24(12):7072–7. doi: 10.1111/jcmm.15335 (PMC7299696; doi:10.1111/jcmm.15335)
Supplement: Supplementary file 6 — Supplementary Material [file JCMM-24-7072-s006.docx]

**Materials and methods**

**Participating Cohorts**

This study was approved by the Institutional Ethics Committee of Nanjing Medical University. All the subjects were recruited from the affiliated hospitals of Nanjing Medical University between 2012 and 2015. Study cohort included women with confirmed RPL, defined as more than 3 consecutive pregnancy losses of undetermined etiology. Characteristics of all participants is given in Supplementary data, Table S1. We excluded patients with abnormal karyotype, infection, endocrine disorders, thyroid dysfunction or abnormal uterine anatomy of RPL. The controls group randomly selected women who underwent legal termination of an apparently normal early pregnancy at the same hospital during the same period. A questionnaire was used to collect clinical and characteristic information, such as personal information, lifestyle factors and medical history. Villi samples were isolated from products of conception (POC) under a dissecting microscope at the time of dilation and curettage and dissected into freezing tubes, transported in liquid nitrogen to the laboratory and stored at -80°C immediately after being washed thoroughly. Tissues were collected with written consent obtained prior to surgery. All activities involved in this study were done under full compliance with government policies and the Helsinki Declaration. All experiments protocol was approved by the Institutional Review Board of Nanjing Medical University prior to the study.

**DNA Isolation and Bisulfite Conversion**

DNA from villi tissues were extracted with the DNeasy Blood & Tissue Kit (Qiagen, Catlog number. 69506) according to the manufacturer’s instructions and quantified with Nanodrop. 1μg DNA was used for bisulfite conversion with the EZ DNA methylation kit (Zymo Research, Catlog number. D5002) using the alternative incubation conditions recommended for the Illumina MethylationEPIC arrays. Before proceeding with methylation profiling, we quantified concentrations of the bisulfite-treated DNA samples using a NanoDrop. Successful conversion was verified by control PCR reactions with a primer set specific for bisulfite-converted DNA, and a primer set for unconverted DNA.

**Genome-wide Methylation Profiling**

Infinium MethylationEPIC BeadChips were used for the determination of methylation levels of more than 850,000 CpG sites. 5 μl of each eluted bisulfite-treated DNA sample was processed according to the protocol supplied by Illumina. The BeadChips were scanned with the Illumina HiScan SQ scanner and raw data were imported to the GenomeStudio to extract the intensities. Probes located on the sex chromosomes and those that had a detection *p* value of greater than 0.01 in one or more samples were removed. We also excluded probes that mapped to more than one location in a bisulfite-converted genome or overlapped with the location of known SNPs, leaving 758, 425 CpGs. Methylation data were processed using the ChAMP package [1]. The signal intensities for the methylated and unmethylated states were normalized using the beta-mixture quantile normalization (BMIQ) algorithm[2]. At each CpG site, the methylation level was reported as a β value and range from 0 (unmethylated) to 1 (methylated).

**DNA methylation data analysis**

Differentially methylated probes (DMPs) were calculated using a linear model by limma package which was implemented in ChAMP [1]. Differentially Methylated regions (DMRs) were computed by Bumphunter, which could firstly cluster all probes into small regions and apply random permutation method to find DMRs [3]. In this study, we chose to identify DMRs as 1 kb gap containing more than 5 probes. Functional annotation analysis of DMRs was performed using HOMER[4], linking DMRs to the nearest genes. Gene ontology analysis was done by DAVID[5, 6].

**RNA Sequencing and analysis**

RNA was extracted from the matched samples used for DNA methylation analysis by RNeasy Kits (Qiagen, Catlog number. 75144) and treated with DNase I (Thermo Fisher Scientific, Catlog number.AM2222) according to standard protocols. RNA-sequencing was done in Genesky using TruSeq Stranded mRNA Library Preparation (Genesky). Briefly, intact RNA was fragmented, end repaired, adapter ligation and PCR amplified following the Illumina protocol. Libraries were sequenced by Illumins Hiseq 2000. After quality control, sequence data were processed with STAR to generate read alignments with hg19[7]. Raw read counts for annotated genes were obtained with featureCounts with default settings[8], normalized and analyzed using DEseq2 [9]. Realtime PCR was used to validate the RNA-seq data. All primers were listed in Supplementary data, Table S2A.

**Bisulfite-sequencing PCR**

Bisulfite treatment was conducted using the EZ DNA methylation kit (Zymo Research, Catlog number.D5002) according to manufacturer's instructions. The bisulfite primers for the *PRDM1* and *ARID5A* were listed in Supplementary data, Table S2B. PCR condition is as follows: 95°C in 10 minutes (1 cycle); 95°C in 30 seconds, 60°C in 30 seconds and 72°C in 40 seconds (40 cycles); 72°C in 10 minutes (1 cycle). Amplified products were purified using the QIAquick gel extraction kit (Qiagen, Catlog number.28706) and cloned into the pMD19-T Vector (Takara, Catlog number.6013). Individual clones were sequenced by ABI Prism 3730xl Genetic Analyzer (Thermo Fisher Scientific). Approximately 10 different clones from each PCR product were sequenced to analyze the methylation status of the CpG sites (CpGs).

**Luciferase reporter assay**

Functional analysis of *PRDM1* DMR was performed with a dual-luciferase reporter assay. A 896 bp fragment was amplified using forward primer 5’-CGAGCTCATGAATCAGCGCATTTTAATCTTC-3’ and reverse primer 5’-CCAAGCTTTATGTTGTTCCTTAAAGGCAACG-3’. The forward/reverse primer contained a SacI/HindIII recognition site. The sequence of the fragment was checked by sequencing. Following double digestion of pGL3-basic vector and *PRDM1* DMR insert with SacI/HindIII (NEB, Catlog number. R0156V and R0104V), the insert was ligated into the vector. CpG Methyltransferase (M.SssI) (NEB, Catlog number.M0226V) was to methylate the fragment. Methylation of fragment was confirmed with the methylation sensitive enzymes HpaII (NEB, Catlog number. R0171V). Both methylated or unmethylated vectors were co-transfected with Renilla control vector in HTR-8/SVneo, JEG-3 and 293T using Lipofectamine 2000(Thermo Fisher Scientific, Catlog number.11668019). Twenty-four hours after transfection, cells were collected to measure the luciferase activity with a Dual-Luciferase Reporter Assay System (Promega, Catlog number. E1910) and the Renilla vectors were used as normalizing control. All experiments were performed in triplicate independently.

**Cell cycle, cell apoptosis and cell migration**

Human *PRDM1* cDNA sequence was synthesis by Realgene biotechnology company and inserted in expression vector pcDNA3.1 (+). The recombinant plasmid was confirmed by endonucleases digesting and DNA sequencing. Two cell lines (HTR-8/SVneo cells and JEG3 cells) were employed for the function analysis *in vitro*. HTR-8/SVneo cell line was cultured in 1640 medium (Gibco, Catlog number.22400089). JEG-3 cell line was cultured in MEM medium (Gibco, Catlog number.12571063) and 293T cell line was cultured in DMEM medium (Gibco, Catlog number.10569010), with 10% fetal bovine serum (FBS) (Gibco, Catlog number.10099133), 100 U/ mL penicillin and streptomycin (Gibco, Catlog number.15140122) at 37°C under 5% CO_2_. Cells were transfected with 4 μg of pcDNA-PRDM1 or pcDNA-Control using Lipofectamine 2000 (Thermo Fisher Scientific, Catlog number. 11668019). After 24 h transfection, cells were stained with propidium iodide (PI) and annexin V for 30 min, then analyzed by FACS (BD Biosciences) to quantify the cell apoptosis or cell cycle. As for cell migration, after 24 h transfection, cells were dyed with crystal violet staining solution (Beyotime, Catlog number. C0121) and photographed under 20×magnification. Each experiment was performed in triplicated independently.

**Chromatin immunoprecipitation (ChIP)**

ChIP method was descripted previously [10]. Briefly, HTR-8/SVneo cells were treated with 1% formaldehyde in PBS for 10 min at room temperature. Cross-linking was terminated by addition of glycine. Cells were lysed and sonicated using a Bioruptor (Diagenode) to generate short fragments for immunoprecipitation. The chromatin was subjected to immunoprecipitation with 1μg of GATA2 (Abcam, Catlog number. ab22849) and FOXA1 (Cell Signaling Technology, Catlog number. 58613) antibody and incubated overnight. After incubating with either protein A or G (Millipore, Catlog number. 17-10086) beads for 2h, the samples were washed with the following low salt buffers, high salt buffers, LiCl buffers and TE buffer. The protein–DNA complexes were reverse crosslinked at 65 °C overnight with Proteinase K. For ChIP-qPCR, primers were listed as follow: Forward primer: 5’-TTCTGGTGTTTACCGCCTGT-3’, Reverse primer: 5’-CTTTTCCTTACTCACGTACCGACT-3’.

**Statistical Analysis**

For DNA methylation and RNA-seq, all statistical tests were conducted in R (version 3.1.1). For DNA methylation and gene expression validation, all data are expressed as the mean ± standard deviation based on at least three independent experiments and student’s t-test (Graphpad) was adopted to estimate the significance of the differences between RPL patients and control groups. The cutoff was 0.05.

**References**

[1]. **Tian Y, Morris TJ, Webster AP, et al.** ChAMP: updated methylation analysis pipeline for Illumina BeadChips. *Bioinformatics*, 2017; 33:3982-4.

[2]. **Teschendorff AE, Marabita F, Lechner M, et al.** A beta-mixture quantile normalization method for correcting probe design bias in Illumina Infinium 450 k DNA methylation data. *Bioinformatics*, 2013; 29:189-96.

[3]. **Jaffe AE, Murakami P, Lee H, et al.** Bump hunting to identify differentially methylated regions in epigenetic epidemiology studies. *Int J Epidemiol*, 2012; 41:200-9.

[4]. **Heinz S, Benner C, Spann N, et al.** Simple combinations of lineage-determining transcription factors prime cis-regulatory elements required for macrophage and B cell identities. *Mol Cell*, 2010; 38:576-89.

[5]. **Huang DW, Sherman BT, Lempicki RA**. Systematic and integrative analysis of large gene lists using DAVID bioinformatics resources. *Nat Protoc*, 2009; 4:44-57.

[6]. **Huang DW, Sherman BT, Lempicki RA**. Bioinformatics enrichment tools: paths toward the comprehensive functional analysis of large gene lists. *Nucleic Acids Res*, 2009; 37:1-13.

[7]. **Dobin A, Davis CA, Schlesinger F, et al.** STAR: ultrafast universal RNA-seq aligner. *Bioinformatics*, 2013; 29:15-21.

[8]. **Liao Y, Smyth GK, Shi W**. featureCounts: an efficient general purpose program for assigning sequence reads to genomic features. *Bioinformatics*, 2014; 30:923-30.

[9]. **Love MI, Huber W, Anders S**. Moderated estimation of fold change and dispersion for RNA-seq data with DESeq2. *Genome Biol*, 2014; 15:550.

[10]. **Qin Y, Roberts JD, Grimm SA, et al.** An obesity-associated gut microbiome reprograms the intestinal epigenome and leads to altered colonic gene expression. *Genome Biol*, 2018; 19:7.
